# The Potential Influence of AI on Population Mental Health

**DOI:** 10.2196/49936

**Published:** 2023-11-16

**Authors:** Catherine K Ettman, Sandro Galea

**Affiliations:** 1 Department of Health Policy and Management Johns Hopkins Bloomberg School of Public Health Baltimore, MD United States; 2 Office of the Dean Boston University School of Public Health Boston, MA United States

**Keywords:** mental health, artificial intelligence, AI, policy, policies, population health, population, ChatGPT, generative, tools, digital mental health

## Abstract

The integration of artificial intelligence (AI) into everyday life has galvanized a global conversation on the possibilities and perils of AI on human health. In particular, there is a growing need to anticipate and address the potential impact of widely accessible, enhanced, and conversational AI on mental health. We propose 3 considerations to frame how AI may influence population mental health: through the advancement of mental health care; by altering social and economic contexts; and through the policies that shape the adoption, use, and potential abuse of AI-enhanced tools.

## Introduction

**Figure 1:**
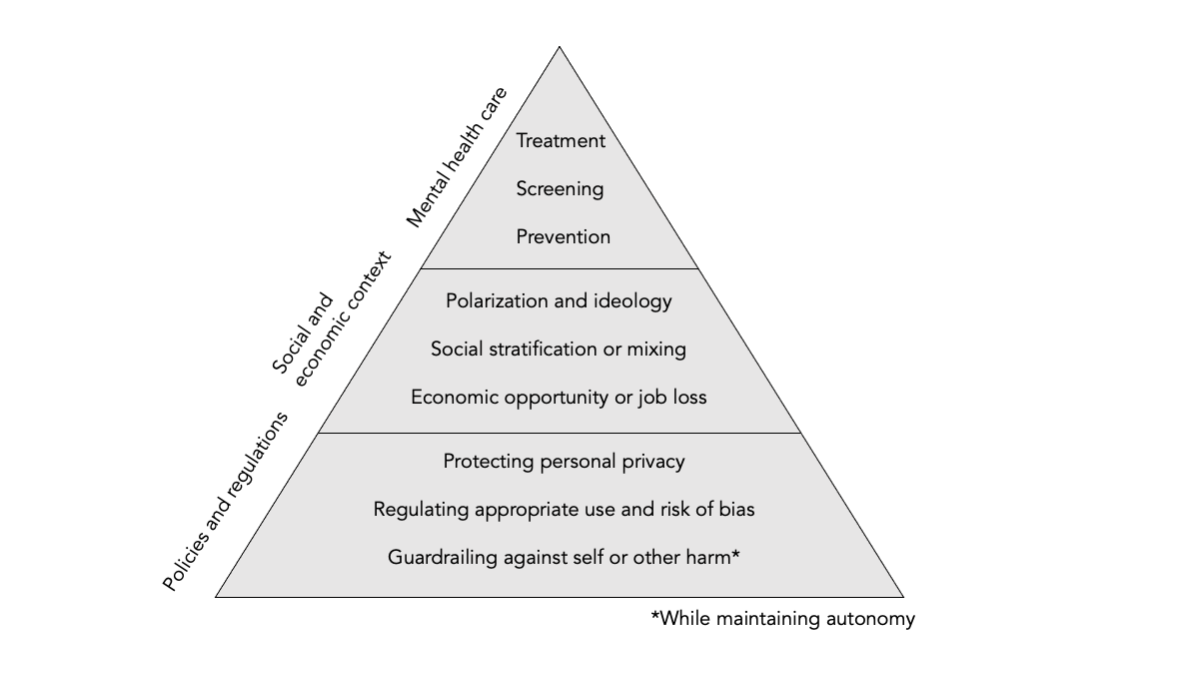
Influence of artificial intelligence on population mental health.

The widespread incorporation of artificial intelligence (AI) in daily use has sparked a global dialogue about the potential benefits and risks of AI on human well-being. Specifically, there is an increasing urgency to anticipate and address the potential impact of widely accessible, enhanced, and conversational AI on mental health. We propose 3 points to consider when determining how AI may influence population mental health: through the advancement of mental health care; by altering social and economic contexts; and through the policies that shape the adoption, use, and potential abuse of AI-enhanced tools (Figure 1).

### Prevention, Screening, and Treatment of Mental Health Disorders

With over 970 million people living with a mental disorder worldwide [1], as well as a shortage of accessible care for many people, leveraging tools such as artificial intelligence (AI) could influence mental health through prevention and treatment. AI-enabled tools can prevent more severe mental illness from developing by identifying higher-risk populations that lead to quicker intervention. AI can detect, assess, and predict stress [2]. For example, AI can process natural language from electronic health records to detect early cognitive impairment [3] or child maltreatment [4], which can have effects on mental health across the course of one’s life.

In addition to preventing mental health challenges through more effective and rapid screening, AI has the potential to improve access to mental health care [5]. One could imagine a world where AI serves as the “front line” for mental health, providing a clearinghouse of resources and available services for individuals seeking help. In addition, targeted interventions delivered digitally can help reduce the population burden of mental illness, particularly in hard-to-reach populations and contexts, for example, through stepped care approaches that aim to help populations with the highest risk following natural disasters.

While AI has promise in terms of early identification of risk and in triaging and treating large volumes of patients, significant flaws exist in using AI for this purpose [6], including bias that may lead to inaccurate assessment and perpetuation of stereotypes. AI efforts to improve risk prediction thus far have been met with mixed results, such as suicide risk prediction by AI being no better than simpler models [7]. The recent improvements in AI technology, however, suggest that as AI improves, it can rapidly become more useful to identify risk for personalized interventions [5]. While some efforts are attempting to leverage AI to deliver mental health care, such as in the form of responsive chatbots, there remains a gulf between vision and implementation—as well as understanding the long-term consequences of replacing human compassion, judgment, and experience with AI-generated responses.

### Social and Economic Contexts That Shape Mental Health

More foundationally, AI may shift or exacerbate differences in the distribution of assets, which serve as a buffer against mental health challenges. Mental health is sensitive to economic and social contexts. First, it is possible that AI may transform or modify existing economic contexts, such as distributions of wealth and employment, which both protect mental health. Unemployment is associated with adverse mental health outcomes long after initial job loss occurs [8]. Potential loss of jobs that may follow AI replacement of specific tasks and industries could lead to psychological sequelae, particularly among workers more vulnerable to job loss, borne disproportionately by populations with fewer assets [7]. In this way, AI could widen existing economic gaps between groups and exacerbate mental health inequities [9,10], thereby fulfilling cumulative inequality theory [11]. Alternatively, AI may benefit mental health through the creation of new entrepreneurial opportunities and access to capital previously unavailable.

Second, the use of AI and generative AI in particular, with human-like responses, may shift how people interact with each other. Meaningful social connections and social support serve as protective mechanisms against diminished health, and AI may shift how people interact with each other. AI may lead to greater polarization and extremism as users consume curated information and may lead to further breakdown of social networks [12] and ties that bond and protect mental health.

### Policy, Regulation, and Guardrails

The policy environment we live in, along with the values that drive our policies, will inform how AI can influence mental health. AI may create opportunities to rapidly synthesize seemingly unlimited information about individuals; if used maliciously, these tools can cause harm to the health of populations. Three considerations, therefore, will be important in this area as we consider how AI may influence population mental health.

First, policies, standards, and regulations should consider how to safeguard sensitive patient information and individuals’ privacy. Given rapidly evolving technology, services, and functions, regulation has not yet kept up with the potential use and misuse of targeted data. Particularly in the case of sharing sensitive mental health data, it will be important to ensure that patients are protected from exposure to malefactors who can exploit their mental health status. While the Health Insurance Portability and Accountability Act (HIPAA) protects digital patient health information in certain settings, it does not extend to new health ecosystems such as the medical internet of things [13] and mobile health (mHealth) applications that collect copious data about individuals and their environments. As the landscape of mental health care and well-being evolves, policies to protect privacy will need to evolve. While there may be benefits to highly accurate data, such as faster arrival of support following suicide and crisis lifeline calls [14], costs include lack of patient privacy and potential abuse by bad actors.

Second, alignment on values and implementation of policy to reduce the influence of bias in AI will be critical to ensure that existing gaps are not exacerbated and that groups are not targeted, mistreated, or maligned intentionally or unintentionally. A growing awareness of the importance of algorithmic fairness has prompted discussion on the appropriate use of AI and machine learning; in the absence of thoughtful intervention, existing algorithms could perpetuate bias and heighten health disparities across groups [15]. Given a history of stigma around mental health in particular, alignment by stakeholders across sectors on the values and sensitivities of using AI broadly will be needed to prevent the exacerbation of stigma and mental health disparities.

Third, guardrails around AI-generated responses can prevent harm. Suicide attempts are more successful when the means used are more lethal; it is possible that users could leverage AI to learn more quickly about self-harm or harming others. Ensuring that AI has built-in guardrails to prevent the proliferation of lethal means and to instead leverage resources to create a pathway to treatment may help to prevent unfavorable outcomes of AI-human engagement.

### Conclusion

While AI may pose potential risks and benefits to human mental health, the mechanism by which they occur is through the real world. Mental health and physical health are experienced in real life. Perhaps the best way to prepare for the oncoming changes that new tools will bring will be to ensure that even as we develop new digital tools, we continue to invest in the basic infrastructure, assets, and social connections that we know protect mental health—and make human life worth living.

## Abbreviations

AI: artificial intelligence
HIPAA: Health Insurance Portability and Accountability Act
mHealth: mobile health
